# A macroeconomic assessment of the impact of medical research expenditure: A case study of NIHR Biomedical Research Centres

**DOI:** 10.1371/journal.pone.0214361

**Published:** 2019-04-10

**Authors:** Joel B. E. Smith, Keith Channon, Vasiliki Kiparoglou, John F. Forbes, Alastair M. Gray

**Affiliations:** 1 Health Economics Research Centre, Nuffield Department of Population Health, University of Oxford, Oxford, United Kingdom; 2 NIHR Oxford Biomedical Research Centre, Oxford University Hospitals NHS Foundation Trust, John Radcliffe Hospital, Oxford, United Kingdom; 3 British Heart Foundation Centre of Research Excellence, Division of Cardiovascular Medicine, University of Oxford, Oxford, United Kingdom; 4 Wellcome Trust Centre for Human Genetics, University of Oxford, Oxford, United Kingdom; 5 Nuffield Department of Primary Care Health Sciences, University of Oxford, Oxford, United Kingdom; 6 Graduate Entry Medical School, University of Limerick, Limerick, Ireland; University of Mississippi Medical Center, UNITED STATES

## Abstract

Quantifying the value of investment in medical research can inform decision-making on the prioritisation of research programmes. Existing methodologies to estimate the rate of return of medical research are inappropriate for early-phase translational research due to censoring of health benefits and time lags. A strategy to improve the process of translational research for patient benefit has been initiated as part of the UK National Institute for Health Research (NIHR) investment in Biomedical Research Centres (BRCs) in England. By providing a platform for partnership between universities, NHS trusts and industry, successful BRCs should reduce time lags within translational research whilst also providing an impetus for local economic growth through industry collaboration. We present a novel contribution in the assessment of early-phase biomedical research by estimating the impact of the Oxford Biomedical Research Centre (OxBRC) on income and job creation following the initial NIHR investment. We adopt a macroeconomic assessment approach using Input-Output Analysis to estimate the value of medical research in terms of income and job creation during the early pathway towards translational biomedical research. Inter-industry linkages are assessed by building a model economy for the South East England region to estimate the return on investment of the OxBRC. The results from the input-output model estimate that the return on investment in biomedical research within the OxBRC is 46%. Each £1 invested in the OxBRC generates an additional £0.46 through income and job creation alone. Multiplicative employment effects following a marginal investment in the OxBRC of £98m during the period 2007-2017 result in an estimated additional 196 full time equivalent positions being created within the local economy on top of direct employment within OxBRC. Results from input-output analyses can be used to inform the prioritisation of biomedical research programmes when compared against national minimum thresholds of investment.

## Introduction

Economic assessments of the impact of medical research aim to capture the direct health benefits to patients, the health system and wider economy. Direct health benefits following investment in medical research include improvements in patient treatment and diagnosis, advancement of scientific knowledge as well as process changes associated with clinical decision making and health care delivery. Quantifying the value of medical research is an important feature of translational research to inform decision-making on the prioritisation of research programmes. The aim of this paper is to outline a methodology for valuing the return on investment of early-phase translational biomedical research. Existing methodologies on medical research impact are constrained by a paucity of data on direct health benefits during early-phase research as well as time lags associated with the translational research process. We use income and job creation as surrogate outcomes for the economic value of medical research during early-phase biomedical research with censored health benefits.

The National Institute for Health Research (NIHR) established the first phase of Biomedical Research Centres (BRCs) in England in 2007 for an initial 5 year period, with the second round of funding initiated in 2012. In the Chancellor’s spending review in the UK in late November 2015, George Osborne protected funding for the third phase of competitive funding for BRCs. The third phase commenced for successful BRCs in spring 2017 as part of the UK Government’s strategy to improve the process of translational research for patient benefit. It is, therefore, timely to evaluate the economic impact of the Oxford BRC (OxBRC). By providing a platform for partnership between universities, NHS trusts and industry, successful BRCs should reduce time lags within translational research whilst also providing an impetus for local economic growth through industry collaboration.

A number of methodologies exist for measuring research impact (See [1–3] for recent reviews). The Payback Framework developed by Buxton and Hanney [4] represents one prominent approach which has been widely applied to estimate the impact of medical research. A key feature of the framework is a non-linear seven stage research process with linkages between research and wider social, political and economic environments. An intuitive appeal of Buxton and Hanney’s payback framework is the ability to capture the full life-cycle of biomedical research from initial research idea inception (Stage 0) to measurement of health, economic and societal benefits (Stage 6). Extensions of the framework have been adopted by Hanney et al. [5] and Wooding et al. [6] for early phase biomedical research. One limitation of the extensions of the Framework for early phase biomedical research is the time lag associated with translational biomedical research. Morris, Wooding and Grant [7] consider the evidence of the frequently reported 17 year time lag of translational research and note the limitations of using a reported average time lag due to substantial heterogeneity. Although a motivation for investment in BRCs is to streamline the translational research process, dissemination of outputs from existing research programmes across BRCs may not have fully matured in a clinical environment within that time period. Evidence to support estimated rates of return within the existing literature are dependent on large time-series data in excess of 20 years to ensure appropriate measurement of both clinical and wider economic outcomes (See, for example, Glover et al. [8]). A central question of this paper is how to measure the returns on medical research when health benefits are yet to be realised as part of early-phase research programmes.

The pathway to translational research is commonly characterised as a two stage process. Type-I translational research is concerned with product development by generating the evidence base from in-vitro pre-clinical testing through to potential implementation in routine clinical care. Type-II translational research focuses upon the implementation stage of products which have shown clinical promise as demonstrated by the Type-I empirical evidence. A microeconomic assessment of changes in health represents a natural starting point for estimating the value of medical research. This micro or “bottom-up” approach aims to first identify the health benefits to the patients as well as isolating linkages with the private sector to capture wider spillover effects to the economy. A bibliometric analysis represents one methodology for very late stage Type-I as well as Type-II translational research outputs. This approach may also synthesise the bibliometric analysis with qualitative approaches with key personnel to elicit achievements and linkages with industry and policy to offer a more comprehensive assessment of research impact. It is clear that this microeconomic approach is problematic for early-phase biomedical research in which health benefits are yet to be realised. Other important considerations can be identified and measured during intermediate steps on the pathway to translational research in light of the role of BRCs as a stimulus for local economic growth. This paper presents a methodology for estimating the economic value of medical research during Type-I biomedical translational research. Using NIHR Oxford Biomedical Research Centre (OxBRC) as an exemplar, we outline the appeal of input-output model multipliers to estimate income and job creation.

To capture the value of early phase biomedical research, this paper will focus exclusively on income and job creation following NIHR investment in the OxBRC. To ensure estimates on the return on investment avoid double-counting, we exclude subsequent public research funding secured following the introduction of the OxBRC. Buxton et al. [9] estimate that the internal rate of return of UK cardiovascular disease research to be 39% during the period 1975 to 2005. Only 9% of the internal rate of return was attributable to health benefits with the remaining 30% derived from wider economic effects. The magnitude of the economic returns relative to the health benefits provides motivation for the focus on income and job creation during the intermediate stages of Type-1 translational biomedical research. Raftery et al. [10] categorises the existing empirical literature on research impact into 20 different methodologies with varying definitions of impact and scope. Direct comparisons across methodologies used in the existing literature are constrained as inputs and outputs on the return on investment are not commensurate. We present a methodology for measuring research impact which ensures input and output data are commensurate across research programmes and disease areas. By ensuring all outputs are a multiplicative effect of all inputs, the methodology addresses concerns about macroeconomic assessments of research impact, such as double-counting and over inflated estimates of return on investment.

## Materials and methods

### Economic model and statistical methods

We propose a macroeconomic assessment or “top-down” approach using Input-Output Analysis to estimate the value of medical research in terms of income and job creation during the early pathway towards translational biomedical research. Input-output models are simplifications of a representative economy and can be used at any spatial level from an individual industry to the global economy. Leontief [11, 12] outlined the appeal of Input-Output analysis as a matrix of supply and consumption dependencies across industries within an economy. The system of equations for the model economy can be expressed in matrix form as:

$$\begin{array}{c} x=\text{Ax}+y \end{array}$$ (1)

where **x** represents a column vector of sectoral supply of goods and services, **y** represents a column vector of inter-industry use of goods and services to produce an output for the respective industry and **A** is a technology matrix, in this case, derived from the inter-industry transaction table for the South East England economy. The solution matrix following a vector of industry changes in demand from an income injection, **y**, which ensures inter-industry demand and supply are equalised, can be expressed as:

$$\begin{array}{c} x={\left(I-A\right)}^{-1}y \end{array}$$ (2)

where **I** represents the identity matrix, (**I − A**)^−1^ is the inverse of (**I − A**) and economic coefficients for the input-output model are derived by (**I − A**)^−1^ [13]. The broad industry classification used to construct the Input-Output model are reported in S1 Table with the corresponding technical coefficients and interdependencies across industrial sectors reported in S2 Table. By constructing a model economy in this way, input-output models aim to assess the interdependence among the different sectors of the economy. The input-output methodology is widely used by government agencies when producing national economic accounts as well as for the assessment of large scale (dis)investment decisions at regional levels. A primary motivation for the use of input-output models is that the effect of a change in demand is not simply confined to an individual industry. Instead, there will often be diffusion and spillover effects to other industries within the economy as well as positive externalities to individuals and households. Although input-output analysis has been previously applied, we present a novel contribution in the assessment of early-phase biomedical research by estimating the economic research impact.

### Outcomes

We aim to assess the economic impact of an investment in early phase biomedical research to the OxBRC. An output multiplier will be obtained from the input-output model to estimate the economic return on investment during the period 2007-2017, synthesising three types of economic effects. The direct effect can be defined as the initial change in income and jobs created following the introduction of the OxBRC. Indirect effects capture the secondary changes in demand across all industrial sectors attributable to the direct effect. Induced effects are the added value from changes in consumption following increased employees’ spending given the indirect effect. The measurement of outcomes is confined to income and job creation following investment in the OxBRC. To derive marginal effects, the initial investment in OxBRC during 2007-2012 is excluded in the input-output analysis. The inclusion of this period would overestimate the rate of return due to the consolidation of existing personnel, equipment and research outputs during the establishment phase of OxBRC. As an illustration of the appeal of input-output methods to quantify the value of early-phase biomedical research, the additional demand generated for the period 2007-2017 is estimated to be of the magnitude of £98m rather than the total direct investment of £157m over the full period. Direct, indirect and induced effects of the OxBRC on job creation are estimated using Type-I and Type-II employment multipliers derived from the input-output model for the South East England economy.

### Data source

The input-output model and derivation of multiplier effects reflect a mixture of regional and national estimates of economic activity. Industry supply and use tables for the UK were obtained from the Office for National Statistics (ONS). Individual industries were aggregated into 10 broad industry classifications to allow more meaningful insights into inter-industry dependency. Variable definitions for each of the 10 industries as well as disaggregated industry components are reported in S1 Table. Technical coefficients for the input-output matrix were calibrated for the South East England region (excluding London) to ensure estimates of impact were proportional to the local economy. Gross value added per capita for the Oxfordshire region were obtained from the Office for National Statistics (Nomis). Employment and financial data from the OxBRC were used to deduct the consolidation period during Phase 1 (2007-2012) of the BRC but the estimated return on investment from the input-output analysis is based on the full implementation period (2007-2017) to provide provisional estimates of impact.

## Results

**Fig 1:**
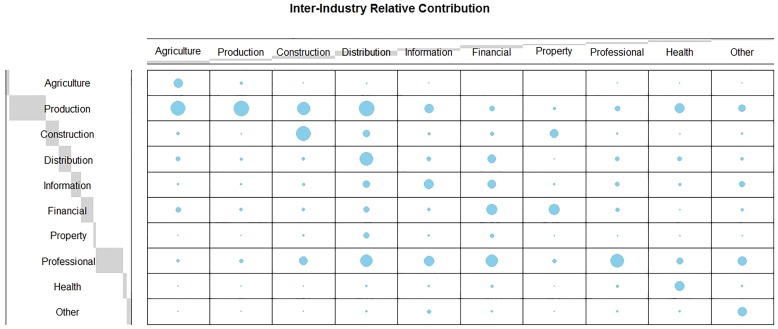
Supply and use matrix for the South East England regional economy. A balloon plot for the relative contribution of each industry to the wider regional economy.

Fig 1 presents the transaction table of sales and payments for the South East England regional economy. Each column captures the inter-industry relationship following a change in demand in each of the 10 industrial sectors. The size of each bubble is weighted by the relative industry’s contribution in the production of those goods and services. In this setting, the larger the bubble within each column the greater the dependency on that industrial sector as a primary supplier in the production process. Each industry is dependent on other sectors of the local economy when producing goods and services. Multiplicative effects of a change in demand within an individual sector in the local economy occur through inter-industry collaboration as well as supply from other firms in order to produce the goods and services. Production, professional service and other health industries are the largest beneficiaries in the production of goods and services within the health industry. Each row captures the relative magnitude of sales across other industries within the local economy. For example, goods and services produced within the health industry are predominately purchased by other firms within the health industry whereas the production industry relies on the agriculture, construction, distribution as well as other firms within the production industry.

**Fig 2:**
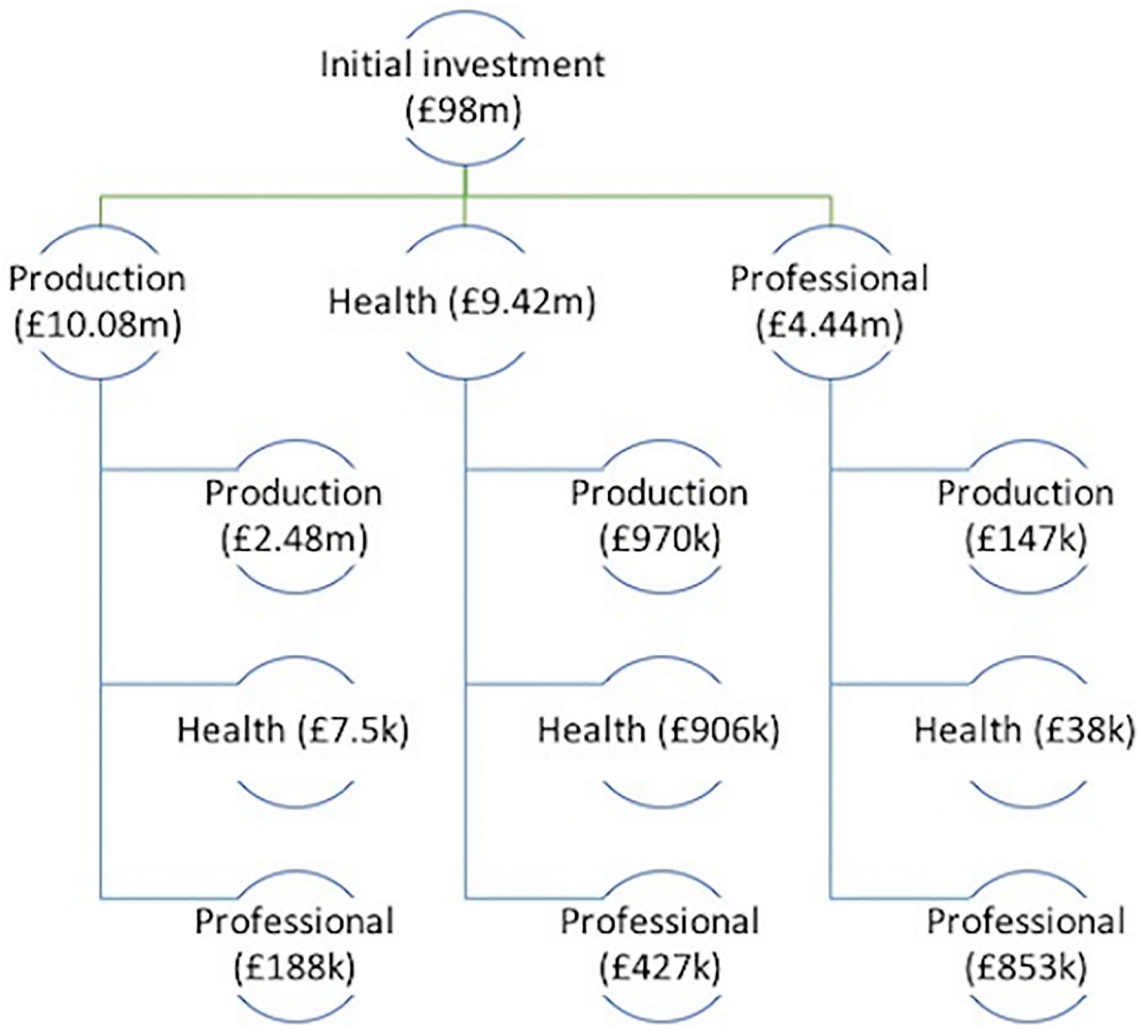
Diffusion and inter-industry spillover effects following the initial marginal investment in OxBRC for selected key industries. A schematic of the diffusion of partial economic effects for key industries following an initial investment in the regional health sector.

The results from the input-output model estimate that the rate of return on investment in biomedical research within the OxBRC is 46%. That is, each additional £1 invested in the OxBRC generates £0.46 through income and job creation alone. A schematic of the diffusion across the 10 industries following an initial £98m investment in the health sector for the OxBRC would be unwieldy and difficult to interpret. As a result, Fig 2 illustrates the results for key beneficiary industries of the health sector for two iterations of changing demand and supply across the sectors. An initial £98m injection into the health industry generates a £10.1m, £9.4m and £4.4m increase in the production, health and professional service industries, respectively. The first round stimulus occurs as goods and services produced by the production, professional service sector and other health industries are used to provide goods and services within the health sector. Second round multiplicative effects occur as demand for goods and services within the beneficiary industries also increases following the first round direct injection.

The £10.1m injection into the production sector increases supply for other firms of the production industry by a further £2.5m, £7,500 in the health sector and £188,000 in the professional services industry. The small magnitude for the health sector is supported by Fig 1 in which the production industry is not dependent on health when producing goods and services. A cyclical process of inter-industry demand and supply filters through the local economy until an equilibrium is reached when demand is saturated across all industries.

The effect of inter-industry changes in demand will also have a direct, indirect and induced effect on employment to meet the increased demand. Multiplicative employment effects derived from the input-output model estimate that 476 [range: 459-492] full time equivalent jobs will be supported and created following the initial £98m investment in OxBRC within the economy. This includes the direct employment within OxBRC, indirect jobs created within beneficiary industries of health and education as well as induced job generation from increases in regional income following industry demand and supply changes. After deducting direct OxBRC employment, the marginal effect on employment is an additional 196 full time equivalent positions created following the investment in OxBRC.

## Discussion

We present a flexible methodology for estimating the economic impact of early-phase biomedical research. The results on income and job creation from the input-output model signify the OxBRCs role as an engine for economic growth within the regional economy. An additional 196 full time equivalent external positions are created across the region through the establishment of the OxBRC as well as £46m generated by stimulating demand across industrial sectors. The input-output model does not capture additional paybacks, such as population health benefits as well as the generation of external research grant income as a result of the marginal effects of NIHR investment in OxBRC. A key rationale for the focus on income and job creation relates to the role of BRCs as providing a platform for clinical research and industry collaboration for patient benefits. Aggregating NIHR funding to BRC institutions as a means of deriving initial estimates of input for an input-output model would result in inflated estimates of impact. Instead, input parameters should be calculated as the marginal effect of NIHR investment after discounting for existing personnel, equipment, research outputs as well as adjustments for local wage inflation and living weighting allowances. The estimated return on investment for OxBRC can be considered a partial assessment and will require updating once health benefits have come to fruition across the full life cycle of the BRC inclusive of translational lagged effects.

The existing payback literature on the value of medical research highlights the disproportional impact of economic benefits in the calculation of rates of return. For example, Buxton et al. [9] estimate that internal rate of return of UK cardiovascular disease research to be 39%, with economic benefits accounting for 76% of the effect over the period 1975 to 2005. Glover et al. [8] estimate the rate of return of cancer research to be 40% with economic effects more than three times the magnitude of the health benefits. The estimated economic return on investment of 46% for OxBRC for income and job creation alone is greater in magnitude than the reported economic and health benefits. One explanation for this result relates to the role of BRCs in streamlining the translational research process and promotion of industry collaboration. The estimated results for the return on investment for OxBRC exceed the UK government’s minimum threshold of 3.5% for investments and, therefore, provide economic justification for the continued investment in OxBRC.

Existing top-down methodologies for valuing medical research may result in over-inflated estimates of impact. Deloitte Access Economics [14] estimate the return on Australian National Health and Medical Research Council investment in cardiovascular disease over the period 2000-2011 to be 509%. Access Economics [15] quantify the returns from all Australian Research and Development (R&D) funding over the period 1992-2005 to be in the region of 110%. The magnitude of some reported returns on investment should be interpreted with caution, and concern that policy makers may be unrealistically primed for returns on medical investment in excess of 100%. Murphy and Topel [16, 17] estimate the return of biomedical R&D in the US in 1995 on health improvements to exceed $2 trillion per year using conservative estimates on the value of a statistical life. We present a unified methodology for valuing early-phase biomedical research that is accessible to policy makers and appropriate for capturing intermediate outcomes on the translational pathway. Although a top-down approach, the estimated return on investment using the input-output methodology is similar in magnitude to other microeconomic methods. This provides a robustness check of the reported estimates and ensures the presented methodology is not biased by double-counting of research activity and impact.

There are potential limitations associated with the input-output methodology. Input-output analyses are dependent on the quality of input parameters and magnitude of multiplier effects. The use of national, regional or local economic estimates on supply and use across industries is not without its challenges and limitations. In the absence of local economic input data, careful consideration about appropriate spatial estimates at the national and regional level is needed to ensure generalisability is maintained at the local level. We use Supply and Use data for the South East England regional economy but exclude London as the industry case-mix for London is considered inappropriate for Oxford and, potentially, unrepresentative of the South East England economy as a whole. Results derived from input-output analyses can be considered to be based on deductive reasoning in which historical data on inter-industry linkages are used to predict changes in the local economy. Within this framework, trade between industries in the past is implicitly assumed to be the best indicator of trade in the future. Although this may not appear too strong an assumption, any structural shifts within an economy may result in current trends that deviate from historical patterns of activity. The result following the 2016 UK European Union (EU) membership referendum would fall into this category depending on the nature of the UK’s EU exit in which pre-2016 economic activity may not provide a useful indicator for a post-EU UK. Data collection on local economic inter-industry linkages would be needed in the absence of robust historical Supply and Use tables.

The OxBRC is used as an exemplar to highlight the appeal and application of input-output analysis with multiplier effects in estimating the value of early-phase biomedical research. Although the present analysis is confined to the South East England regional economy using a linear deterministic input-output model, the methods are flexible enough to be applied in the evaluation across all NIHR BRCs using dynamic or non-linear input-output methods. If, however, the research question is concerned with the evaluation of all medical research undertaken by a particular funding agency then extensions of the input-output methodology would be fruitful. This may include the adoption of a Social Accounting Matrix and General Equilibrium Modelling methodologies.

## Conclusion

Existing top-down methodologies for valuing medical research result in over-inflated estimates while microeconomic assessments are constrained by censored benefits. Input-output analysis overcomes these limitations resulting in unbiased estimates while ensuring that the true value of early-phase biomedical research is greater than the sum of the parts. We exclude subsequent public research funding secured following the introduction of the OxBRC to avoid double-counting as some of this activity would have occurred independent of the expenditure on the OxBRC. The results, therefore, represent conservative estimates on the return on investment. As our results are generated using data for the South East England regional economy, the generalisability to differing spatial contexts should apply the top-down methodology using relevant local data. The estimated results of the return on investment for OxBRC exceed the UK Government’s minimum threshold of 3.5% for investments and, therefore, provide economic justification for the continued investment in OxBRC.

## Supporting information

S1 Table Definition of the 10 broad industry classifications. Supplementary Table defining each of the 10 broad industry classifications. The Table is proudced using the “longtable” package in LaTeX and can be viewed as a single .pdf document.
(PDF)

S2 Table Matrix of technical coefficients and interdependencies. Supplementary Table reporting the technical coefficients for each industry as well as the interdependencies across industrial sectors. The Table can be viewed as a single .pdf document.
(PDF)

## References

[1] Greenhalgh T and Raftery J and Hanney S and Glover M. Research impact: a narrative review. BMC Med. 2016; 14:78 10.1186/s12916-016-0620-8 27211576 PMC4876557

[2] Rivera SC, Kyte DG, Aiyegbusi OL, Keeley TJ, Calvert MJ. Assessing the impact of healthcare research: A systematic review of methodological frameworks. PLoS Med. 2017; 14(8):1–24. 10.1371/journal.pmed.1002370 PMC5549933 28792957

[3] Milat AJ, Bauman AE, Redman S. A narrative review of research impact assessment models and methods. Health Res Policy Syst. 2015; 13:18 10.1186/s12961-015-0003-1 25884944 PMC4377031

[4] Buxton M, Hanney S. How can payback from health services research be assessed?. J Health Serv Res Policy. 1996; 1(1):35–43. 10.1177/135581969600100107 10180843

[5] Hanney S, Grant J, Wooding S, Buxton M. Proposed methods for reviewing the outcomes of research: the impact of funding by the UK’s’Arthritis Research Campaign’. Health Res Policy Syst. 2004; 2(4). 10.1186/1478-4505-2-4 15272939 PMC503400

[6] Wooding S, Hanney S, Buxton M, Grant J. Payback arising from research funding: evaluation of the Arthritis Research Campaign. Rheumatology. 2005; 44(9):1145–1156. 10.1093/rheumatology/keh708 16049052

[7] Morris ZS, Wooding S, Grant J. The answer is 17 years, what is the question: understanding time lags in translational research. J R Soc Med. 2011; 104(12): 510–520. 10.1258/jrsm.2011.110180 22179294 PMC3241518

[8] Glover M, Buxton M, Guthrie S, Hanney S, Pollitt A, Grant J. Estimating the returns to UK publicly funded cancer-related research in terms of the net value of improved health outcomes. BMC Med. 2014; 12(1):99 10.1186/1741-7015-12-99 24930803 PMC4058434

[9] Health Economics Research Group (HERG), Office of Health Economics (OHE), RAND Europe. Medical research–what’s it worth? Estimating the economic benefits from medical research in the UK. London: UK Evaluation Forum; 2008.

[10] Raftery J, Hanney S, Greenhalgh T, Glover M, Young A. Models and applications for measuring the impact of health research: Update of a systematic review for the Health Technology Assessment Programme. Health Technol Assess. 2016; 20(76):1–282. 10.3310/hta20760 27767013 PMC5086596

[11] Leontief W. Quantitative Input and Output Relations in the Economic System of the United States. Rev Econ Stat. 1936; 18(3):105–125. 10.2307/1927837

[12] Leontief W. The Structure of American Economy, 1919-1939, An empirical application of equilibrium analysis. New York, Oxford University Press; 1951.

[13] Miller RE, Blair PD. Input-Output Analysis: Foundations and Extensions. Cambridge, Cambridge University Press; 2009.

[14] Deloitte Access Economics. Returns on NHMRC funded Research and Development. Deloitte Access Economics: Canberra; 2011.

[15] Access Economics. Exceptional Returns: The Value of Investing in Health R&D in Australia II. Canberra: Access Economics; 2008.

[16] Murphy KM, Topel RH. The Economic Value of Medical Research In Measuring the Gains from Medical Research: An Economic Approach. edited by Murphy Kevin M and Topel Robert H. Chicago: Univ. Chicago Press; 2003.

[17] Murphy KM, Topel RH. The Value of Health and Longevity. J Polit Econ. 2006; 114(5): 871–904. 10.1086/508033

