# Correction: A macroeconomic assessment of the impact of medical research expenditure: A case study of NIHR Biomedical Research Centres

**DOI:** 10.1371/journal.pone.0216315

**Published:** 2019-04-25

**Authors:** Joel B. E. Smith, Keith M. Channon, Vasiliki Kiparoglou, John F. Forbes, Alastair M. Gray

The second author’s name is spelled incorrectly. The correct name is: Keith M. Channon.

The correct citation is: Smith JBE, Channon KM, Kiparoglou V, Forbes JF, Gray AM (2019) A macroeconomic assessment of the impact of medical research expenditure: A case study of NIHR Biomedical Research Centres. PLoS ONE 14(4): e0214361. https://doi.org/10.1371/journal.pone.0214361
