# Supplementary material for: A macroeconomic assessment of the impact of medical research expenditure: A case study of NIHR Biomedical Research Centres
Source: PLoS One. 2019 Apr 10;14(4):e0214361. doi: 10.1371/journal.pone.0214361 (PMC6457483; doi:10.1371/journal.pone.0214361)
Supplement: S1 Table — Supplementary Table defining each of the 10 broad industry classifications. The Table is proudced using the “longtable” package in LaTeX and can be viewed as a single .pdf document. (PDF) [file pone.0214361.s001.pdf]

Table S1: Definition of the 10 broad industry classifications.

Industry	Industry Components
Agriculture	Products of agriculture, hunting and related services; Products of forestry,logging and related services; Fish and other fishing products, aquaculture products and support services to fishing.
Production	Coal and lignite; Crude Petroleum And Natural Gas & Metal Ores; Other mining and quarrying products; Mining support services; Preserved meat and meat products; Processed and preserved fish, crustaceans, molluscs, fruit and vegetables; Vegetable and animal oils and fats; Dairy products; Grain mill products, starches and starch products; Bakery and farinaceous products; Other food products; Prepared animal feeds; Alcoholic beverages; Soft drinks; Tobacco products; Textiles; Wearing apparel; Leather and related products; Wood and of products of wood and cork, except furniture; articles of straw and plaiting materials; Paper and paper products; Printing and recording services; Coke and refined petroleum products; Paints, varnishes and similar coatings, printing ink and mastics; Soap and detergents, cleaning and polishing preparations, perfumes and toilet preparations; Other chemical products; Industrial gases, inorganics and fertilisers (all inorganic chemicals); Petrochemicals; Dyestuffs, agro-chemicals; Basic pharmaceutical products and pharmaceutical preparations; Rubber and plastic products; Manufacture of cement, lime, plaster and articles of concrete, cement and plaster; Glass, refractory, clay, other porcelain and ceramic, stone and abrasive products; Basic iron and steel; Other basic metals and casting; Weapons and ammunition; Fabricated metal products, excl. machinery and equipment and weapons & ammunition; Computer, electronic and optical products; Electrical equipment; Machinery and equipment; Motor vehicles, trailers and semi-trailers; Ships and boats; Air and spacecraft and related machinery; Other transport equipment; Furniture; Other manufactured goods; Repair and maintenance of ships and boats; Repair and maintenance of aircraft and spacecraft; Rest of repair, Installation; Electricity, transmission and distribution; Gas, distribution of gaseous fuels through mains, steam and air conditioning supply; Natural water, water treatment and supply services; Sewerage services, sewage sludge; Waste collection, treatment and disposal services, materials recovery services
Construction	Construction
Distribution	Wholesale and retail trade and repair services of motor vehicles and motorcycles; Wholesale trade services, except of motor vehicles and motorcycles; Retail trade services, except of motor vehicles and motorcycles; Rail transport services; Land transport services and transport services via pipelines, excluding rail transport; Water transport services; Air transport services; Warehousing and support services for transportation; Postal and courier services & Accommodation services; Food and beverage serving services
Information	Publishing services; Motion Picture, Video & TV Programme Production, Sound Recording & Music Publishing Activities & Programming And Broadcasting Activities; Telecommunications services; Computer programming, consultancy and related services; Information services
Financial	Financial services, except insurance and pension funding; Insurance and reinsurance, except compulsory social security & Pension funding; Services auxiliary to financial services and insurance services
Property	Real estate services, excluding on a fee or contract basis and imputed rent; Owner-Occupiers' Housing Services; Real estate activities on a fee or contract basis
Professional	Legal services; Accounting, bookkeeping and auditing services; tax consulting services; Services of head offices; management consulting services; Architectural and engineering services; technical testing and analysis services; Scientific research and development services; Advertising and market research services; Other professional, scientific and technical services; Veterinary services; Rental and leasing services; Employment services; Travel agency, tour operator and other reservation services and related services; Security and investigation services; Services to buildings and landscape; Office administrative, office support and other business support services
Health	Education services; Human health services; Residential Care & Social Work Activities

Other	Creative, arts and entertainment services; Libraries, archives, museums and other cultural services; Gambling and betting services; Sports services and amusement and recreation services; Services furnished by membership organisations; Repair services of computers and personal and household goods; Other personal services; Services of households as employers of domestic personnel
