# Supplementary material for: A macroeconomic assessment of the impact of medical research expenditure: A case study of NIHR Biomedical Research Centres
Source: PLoS One. 2019 Apr 10;14(4):e0214361. doi: 10.1371/journal.pone.0214361 (PMC6457483; doi:10.1371/journal.pone.0214361)
Supplement: S2 Table — Supplementary Table reporting the technical coefficients for each industry as well as the interdependencies across industrial sectors. The Table can be viewed as a single .pdf document. (PDF) [file pone.0214361.s002.pdf]

Table 1: Matrix of technical coefficients and interdependencies.

	**Agri.**	**Prod.**	**Con.**	**Dist.**	**Info.**	**Finan.**	**Prop.**	**Prof.**	**Health**	**Other**
**Agriculture**	0.0907	0.0093	0.0013	0.0047	0.0001	0.0000	0.0000	0.0000	0.0002	0.0001
**Production**	0.2274	0.2464	0.1673	0.2348	0.0849	0.0314	0.0085	0.0332	0.1029	0.0530
**Construction**	0.0162	0.0040	0.2243	0.0567	0.0087	0.0178	0.0790	0.0048	00037	0.0076
**Distribution**	0.0275	0.0110	0.0143	0.1898	0.0227	0.0794	0.0023	0.02187	0.0237	0.0153
**Information**	0.0065	0.0056	0.0091	0.0537	0.1009	0.0725	0.0058	0.0271	0.0115	0.0350
**Financial**	0.0320	0.0129	0.0150	0.0381	0.0159	0.1241	0.1235	0.0202	0.0037	0.0112
**Property**	0.0018	0.0009	0.0061	0.0396	0.0049	0.0168	0.0030	0.0026	0.0045	0.0045
**Professional**	0.0135	0.0187	0.0780	0.1583	0.1070	0.1568	0.0200	0.1921	0.0453	0.0849
**Health**	0.0003	0.0007	0.0008	0.0051	0.0058	0.0094	0.0006	0l0085	0.0962	0.0050
**Other**	0.0015	0.0005	0.0002	0.0064	0.0163	0.0073	0.0003	0.0054	0.0064	0.0909

Table note: Argi=Agriculture; Prod=Production; Con=Construction; Dist=Distribution; Info=Information; Finan=Financial Prop=Property; Prof=Professional
